# Liquid Chromatography Quadrupole Time-of-Flight Mass Spectrometry-Based Metabolic Characterization of Mango Ripened by Different Methods

**DOI:** 10.3390/foods13223548

**Published:** 2024-11-07

**Authors:** Jishi Wang, Chaoqi Ren, Jiafu Wang, Jiqiang Fu, Qingchun Yin, Yongping Huang, Zeying He

**Affiliations:** 1 Key Laboratory for Environmental Factors Control of Agro-Product Quality Safety, Ministry of Agriculture, Agro-Environmental Protection Institute, Ministry of Agriculture and Rural Affairs, Tianjin 300191, China; wangjishi@caas.cn (J.W.); wjf199809@163.com (J.W.); 82101225353@caas.cn (J.F.); 2 College of Animal Science and Veterinary Medicine, Tianjin Agricultural University, Tianjin 300384, China; renchaoqi92@163.com; 3 Key Laboratory of Tropical Fruits and Vegetables Quality and Safety, Institute of Food Testing, Hainan Academy of Inspection and Testing, State Administration for Market Regulation, Haikou 570311, China; yinqingchun@163.com

**Keywords:** naturally ripened mango, artificially ripened mango, metabolomics, multivariate analysis

## Abstract

So far, the metabolic differences between tree-ripened and postharvest-ripened mangoes have largely remained unexplored. The aim of this study was to evaluate the chemical composition of nutrient substances in mangoes subjected to different ripening methods. An untargeted metabolomic approach based on ultra performance liquid chromatography coupled to quadrupole time-of-flight mass spectrometry (UPLC-Q-TOF-MS) was carried out to investigate the differences between artificially ripened and naturally ripened mangoes. The principal component analysis results indicate a clear separation between the different treatment groups. Variance analysis, fold change, and orthogonal partial least squares discriminant analysis (OPLS-DA) were employed to find potential markers. In total, 69 metabolites were identified, with significant variations in the abundance of organic acids, vitamins, carbohydrates, and polyphenols closely related to the ripening methods of mangoes. These results contribute to a better understanding of the metabolic changes in mangoes due to different ripening methods, which could be used to assist in evaluating the quality of mango fruit.

## 1. Introduction

Mango (*Mangifera indica* L.), commonly known as the ‘the king of tropical fruit’, is one of the most popular fruits in the word due to its pleasant aroma, unique flavor, and rich nutritional profile [1,2]. As is well known, mango is rich in various bioactive compounds, including vitamins, organic acids, carbohydrates, and polyphenols [1]. Mango production and trade volumes are gradually expanding, with the global production reaching 63.11 million tons in 2022 [3]. Currently, India is the largest producer of mangoes, followed closely by China. Mango is a typical climacteric type of fruit, generally harvested at the early stage of maturity (when physiologically developed) and then ripened to achieve the desired flavor and texture [4,5]. Tree-ripened mango fruits have a shorter shelf life and deteriorate rapidly, which restricts their storage, transportation, and sales [6]. To avoid these issues, fruits are harvested at the pre-climacteric mature green stage, which may directly affect the physicochemical properties and polyphenol biosynthesis of the mango [7]. After harvesting, mangoes continue to ripen, during which their metabolic activities undergo significant changes, including an increased respiration intensity, rapid ethylene production, and the biosynthesis of pigments such as carotenoids and anthocyanins [8].

Ethylene, a key plant hormone, plays a critical role in regulating the postharvest ripening of climacteric fruits [9]. It influences metabolic pathways of ripening at different levels, including metabolite synthesis, signal transduction, and related genes expression, thereby controlling the changes that occur during fruit ripening [10]. Climacteric fruits are typically treated with exogenous ethylene before marketing to promote the rapid synthesis of endogenous ethylene. This accelerates a series of physiological responses associated with fruit ripening, including skin color transformation from green to yellow, reduced flesh firmness, chlorophyll degradation, carotenoid synthesis, conversion of organic acids to amino acids, and the conversion of starch to sugars, thereby achieving a desirable fruit quality for sale to meet consumer demand [8]. In recent years, extensive research has been conducted on the effects of ethylene on the postharvest ripening of mangoes. Ho et al. discovered that treating mangoes with ethylene α-cyclodextrin inclusion complex powder during transportation leads to the release of ethylene gas, which stimulates the continuous production of endogenous ethylene in mangoes and significantly shortens the postharvest ripening time [11]. Razzaq et al. demonstrated that treatment with 2-chloroethylphosphonic acid (ethephon) increases ethylene production, accelerates the respiration rate of ‘Kensington Pride’ mangoes, and promotes fruit ripening [12]. The combination of ethephon and improved atmospheric packaging can enhance mango ripening and the overall fruit quality, such as reducing fruit firmness and acidity while increasing the fruit color index, total soluble solids, and the TSS/acidity ratio [13]. Zaharah et al. demonstrated that treatment with abscisic acid induced enhances in the activity of inocyclopropane-1-carboxylic acid synthase and inocyclopropane-1-carboxylic acid oxidase and stimulated ethylene production, which may contribute to an increased pectinase activity and accelerate the mango softening process [14]. Although the aforementioned studies indicate that the response of postharvest mango fruit to ripening agents involves a series of physiological and metabolic changes, very limited research has been published regarding the changes in endogenous metabolites of mangoes post-ripening based on high-throughput metabolomics.

Metabolomics is a systematic approach that analyzes the profile of small molecule metabolites from a specific cell, organ, or organism to find interesting metabolic trends and elucidate the ultimate response of biological systems to endogenous or exogenous stimuli [15]. Metabolomics has been applied to various research fields, including medicine and diagnosis [16], toxicology [17], environmental sciences [18], and nutrition [19]. The rapid development of this method benefits from mass spectrometry (MS), nuclear magnetic resonance (NMR), and chemometrics-based software. Quadrupole time-of-flight mass spectrometry (Q-TOF/MS) is characterized by high acquisition rates, excellent sensitivity, high resolution, and satisfactory mass accuracy. It enables comprehensive analysis of small molecular metabolites and has become one of the most important tools in metabolomics research [20]. Organic pollutants, including plant growth regulators, act as exogenous interference factors affecting the metabolism of agricultural products, ultimately resulting in changes in nutritional quality. He et al. discovered that citric acid and various lipids were significantly down-regulated in citrus fruits treated with asomate compared to the control using UPLC-Q-TOF/MS-based metabolomic [21]. Untargeted metabolomics and transcriptomics analyses indicate that cyflumetofen alters the nutritional and flavor characteristics of apples by interfering with amino acid, organic acid, polyphenol, and lipid metabolism pathways [22]. Zhang et al. observed that fertilization and pesticide application influence the composition of sugars, amino acids, and organic acids in citrus fruits using proton nuclear magnetic resonance (¹H NMR) metabolomics [23]. The total contents of aldehydes, terpenes, esters, and furanones in strawberries were significantly reduced after treatment with the fungicides boscalid and difenoconazole compared with the control [24].

This work aimed to investigate the metabolite profiles of mango samples with different ripening methods using ultra performance liquid chromatography coupled to quadrupole time-of-flight mass spectrometry (UPLC-Q-TOF-MS), combined with multivariate analysis. Significant markers associated with ripening modes in mangoes were discovered and identified, and their biological significance was explored through comprehensive metabolomic analysis. The information derived from this study provides insights into the effects of after-ripening on mango metabolites and their potential mechanisms.

## 2. Materials and Methods

### 2.1. Chemicals and Reagents

Acetonitrile and methanol were of HPLC grade and were bought from Fisher Scientific (Fair Lawn, NJ, USA). Ammonium acetate and formic acid were purchased from Dikma Technologies (Beijing, China). Water used for all the tests in this research was produced in a Milli-Q system (Millipore, Billerica, MA, USA).

### 2.2. Fruit Material and Treatments

Mango (Mangifera indica L. cv. Tainung No.1) fruits were harvested at mature green stage from an orchard located in Sanya City, Hainan province, China. Mango fruits that are uniform in size, color, and free from defects were selected, subsequently subjected to transient surface disinfection with sodium hypochlorite solution (0.1%, *v*/*v*), rinsed with distilled water, and then dried at room temperature for further experimentation. The disinfected fruits were immersed in distilled water (control, 10 samples), and in ethylene solution at concentrations of 200 mg/L (low, 6 samples), 400 mg/L (medium, 6 samples) and 800 mg/L (high, 6 samples) for 15 min. Afterwards, the treated fruits were air-dried at room temperature, packaged in polyethylene bags, and stored in a climate cabinet (20 °C, 85% RH) until fully ripe. Naturally ripened mangoes (10 samples), sourced from the same orchard, were transported to the laboratory by air on the day of harvest. After being disinfected with sodium hypochlorite solution (0.1%, *v*/*v*), these mangoes were processed alongside artificially ripened mango samples. All fresh samples were immediately frozen with liquid nitrogen, homogenized using an automatic tube mill (IKA, Staufen, Germany), freeze-dried (Christ, Osterode, Germany), and preserved at −80 °C until analysis.

### 2.3. Sample Preparation

A 100 mg sample of freeze-dried mango was weighed into a 10 mL Eppendorf tube, and 2.5 mL of pre-cooled 80% methanol/water (4:1, *v*/*v*) solution was added. The tube was shaken for 15 min, sonicated for 10 min in an ice bath, and then left to stand at −20 °C for 1 h. Following this, it was centrifuged at 14,000× *g* for 10 min. The supernatant was filtered through a 0.22 μm filter (Millipore, Billerica, MA, USA) and subsequently analyzed using UPLC-QTOF/MS.

Quality control (QC) samples were prepared using an equal pooling of all mango samples to ensure that the data were stable and repeatable [25]. During the instrumental analysis, a QC sample was inserted after every five samples and then analyzed.

### 2.4. UPLC-Q-TOF Untargeted Analysis

An Exion LC UPLC system coupled to a Q-TOF mass spectrometer (Triple TOF 7600; Sciex Inc., Foster City, CA, USA) was used for high-resolution mass analysis. Chromatographic separation was achieved on a HSS T3 C18 column (2.1 × 100 mm, 1.8 μm; Waters Corp., Milford, MA, USA) maintained at 40 °C. The mobile phases A and B for positive ion mode were water with 5 mM ammonium formate and 0.1% formic acid and acetonitrile with 0.1% formic acid, respectively. The mobile phases A and B for negative ion mode were water with 5 mM ammonium formate (adjusted to pH 7.5) and acetonitrile, respectively. The flow rate was set at 0.3 mL/min, and the injection volume was 2 μL. For both positive and negative modes, the gradient program was as follows: 0–1.5 min, 3% eluent B; 1.5–11 min, 3–80% eluent B; 11–14 min, 80–97% eluent B; maintained for 4 min; 18–18.1%, 97–3% eluent B; maintained for 2.9 min. The total analytical time for each sample was 21 min. The electrospray ionization (ESI) parameters setting were as follows: ion source (IS), 5500 V for positive mode and −4500 V for negative mode; ion spray probe temperature, 500 °C; nebulizer gas (GS1, N_2_), 55 psi; heater gas (GS2, N_2_), 55 psi; curtain gas (CUR), 35 psi. Mass spectral data were collected in independent data acquisition (IDA) mode, with TOF-MS range set to 100–1250 *m*/*z*) and tandem MS range set to 50–1250 *m*/*z*. In the full-scan analysis, the declustering potential (DP) and collision energy (CE) were set at 80 eV and 35 ± 15 eV, respectively. The dynamic background subtraction was carried out to acquire accurate precursor and product ion mass information.

### 2.5. Data Processing and Statistical Analyses

The peak area extraction and peak identification were carried out using Sciex OS software (Version 2.2.0.5738, Sciex Inc., Foster City, CA, USA). Data acquisition was achieved using the following parameter settings: mass window, 0.02 Da; mass tolerance, 20 mDa; and intensity ≥ 100 counts. The data preprocessing was performed using MetaboAnalyst 6.0 (http://www.metaboanalyst.ca, accessed on 8 August 2024) online software. First, metabolites with missing values greater than 50% were removed, and all missing values were replaced with half of the minimum positive value from the original data, assumed to be equal to the limit of detection. Second, compounds with a relative standard deviation (RSD) > 25% were removed from the QC samples. Finally, all data were normalized to the mean centering and divided by the square root of the standard deviation for each variable. The data obtained from both positive and negative ion modes were combined into a single dataset for subsequent multivariate statistical analyses, including principal component analysis (PCA) and orthogonal partial least squares discriminant analysis (OPLS-DA), to determine the relationship between groups and metabolite profiles. The variable importance in projection (VIP) value was calculated from the OPLS-DA; a VIP > 1 indicated that the metabolite has a significant contribution to the separation of sample groups. Additionally, analysis of variance (ANOVA) and fold change (FC) were performed to filter compounds extracted from the raw data. Compounds with VIP > 1, *p*-values < 0.05, and FC > 2 (or <0.5) were considered potential biomarkers [26]. Our laboratory previously constructed a plant metabolomics database containing over 700 compounds by analyzing standard reference materials. The differential markers were identified by searching this custom-built database using precursor *m*/*z* and MS/MS spectra.

## 3. Results and Discussion

### 3.1. Untargeted Metabolomics Analysis

**Figure 1:**
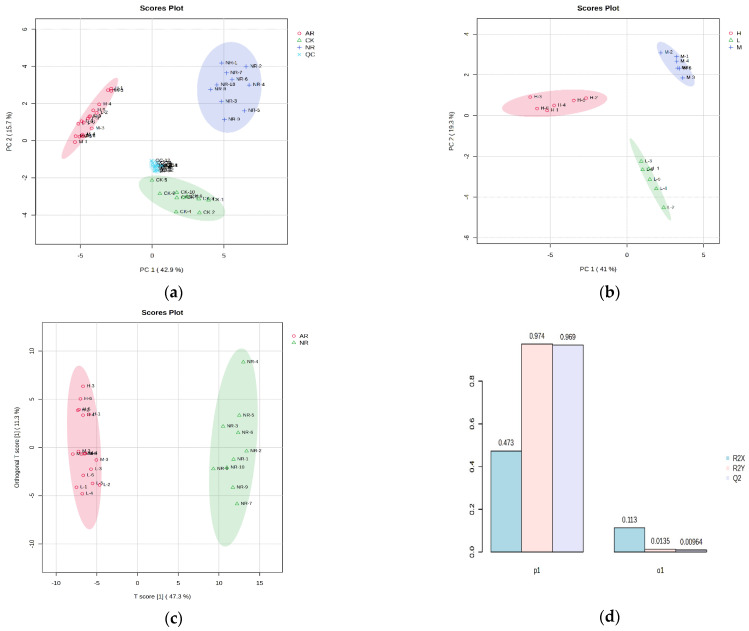
(**a**) PCA score plot of mango samples with different ripening methods (QC, quality control; NR, naturally ripened group; AR, artificially ripened group; CK, control group); (**b**) PCA score plot of mango samples ripened with different ethylene concentrations (H, high concentration; M, medium concentration; L, low concentration); (**c**) OPLS-DA score plot of naturally ripened group and artificially ripened group; (**d**) OPLS-DA model of naturally ripened group and artificially ripened group (one predictive and one orthogonal components). The colored ellipses represent 95% confidence regions for each group.

Metabolites in mangoes were extensively extracted and analyzed using UPLC-Q-TOF/MS. A total of 1965 metabolite ion features were extracted in both positive and negative ion modes. PCA was performed to determine whether mango samples with different ripening methods could be distinguished. The PCA score plot shows that all data points are located within the 95% confidence ellipse, with the separation into distinctive clusters of QC, control (CK), naturally ripened (NR), and artificially ripened (AR) groups (Figure 1a). This finding suggests that the collected data can effectively differentiate among the various samples. The first three principal components (PC1, PC2 and PC3) explain 42.9%, 15.7%, and 10.4% of the variation, respectively. All QC results appear at the center of the PCA score plot, indicating high instrument accuracy and satisfactory reproducibility of the analytical method. It was noted that, when analyzing only the artificially ripened mango samples, the PCA score plot clearly divided the data points into high, medium, and low groups based on their metabolite profiles (Figure 1b). This indicates that the differences in compound abundance between groups are greater than those observed within groups. Consequently, these differential compounds will be the primary focus of our study.

Supervised OPLS-DA was carried out to further investigate the differences in the three groups and determine the optimal features for distinguishing mangoes. The results show that the coefficient of determination (R^2^X) was 47.3%, which explained 97.4% of the variance in the response (R^2^Y) observed between artificially ripened mangoes and naturally ripened mangoes. The Q^2^ (cum) value, obtained through seven-fold cross validation, is 96.9% (>50%) demonstrated that the OPLS-DA model had excellent predictive capacity (Figure 1c,d). The OPLS-DA modes of AR/CK and NR/CK groups produced R^2^(X) values of 41.9% and 32.4%, R^2^(Y) values of 96.3% and 93.6, and Q^2^ (cum) values of 95.6% and 91.6%, respectively (Supplementary Figure S1). Permutation tests were carried out 1000 times to assess data overfitting. As shown in Figure S2, all three models were statistically valid and acceptable (intercepts, R^2^(Y): 0.988, 0.994 and 0.987 (*p* < 0.001); Q2: 0.978, 0.986, and 0.963 (*p* < 0.001)). The above results indicate that the OPLS-DA models are not overfitting and are statistically valid.

### 3.2. Potential Biomarkers Identification

To screen and identify potential biomarkers in mango samples with different ripening methods, we performed three criterions to the pre-normalized data: VIP > 1, *p* < 0.05, and FC > 2 (or <0.5). The results showed that 69 metabolites of interest were screened. Among these 69 metabolites, 11 potential markers were identified and annotated by matching with the MS/MS high-resolution library. The extracted ion chromatogram and fragment-ion information matched against the library of the confirmed metabolites are provided in the Supplementary Material (Figure S3). An additional 58 metabolites were preliminarily identified. The 69 metabolites include 18 amino acids and derivatives, 2 carbohydrates and derivatives, 16 polyphenols, 3 nucleosides, 5 lipid compounds, 9 organic acids, 2 peptides, 4 phospholipids, 3 terpenoids, 3 vitamins with cofactors, and 4 others (Table 1).

**Figure 2:**
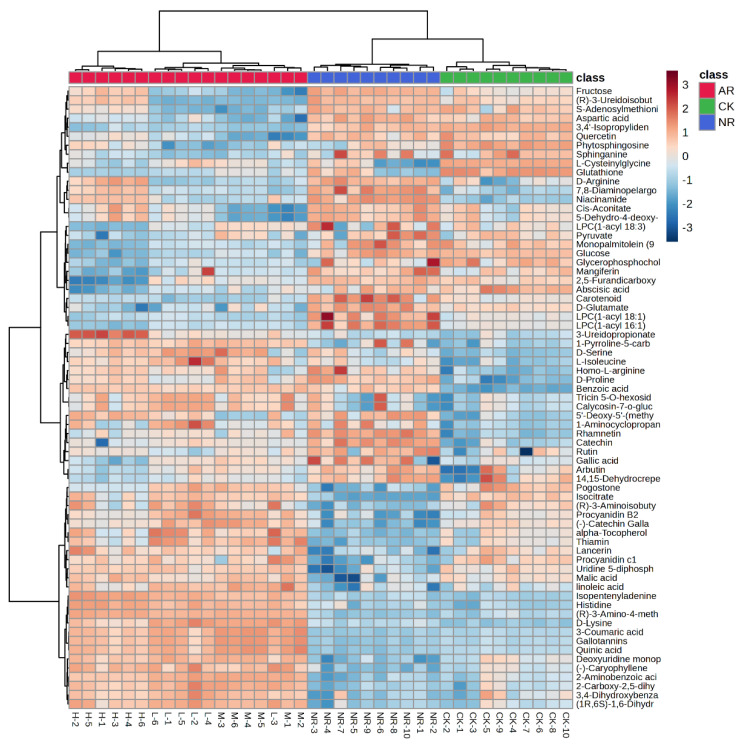
Heatmap of identified metabolites with significant changes from mango samples ripened by different methods.

The 69 compounds abundances for the three groups were loaded into the hierarchical clustering analysis (HCA), and the results were presented as a heatmap. Cases and compounds are represented on the horizontal and vertical axes, respectively. In the heatmap, the correlations between cases were determined independently based on the abundances of metabolites, with no prior assumptions made. As shown in Figure 2, there is a significant clustering phenomenon among the control group, artificially ripened group, and naturally ripened group. It is evident that the metabolite abundances of the NR group and CK group are more similar compared to the AR group, and they are classified within the same major group. Interestingly, the AR group is further branched into three subgroups. The medium and low concentration groups are clustered on the same branch, whereas the high concentration group is on a separate branch in the dendrogram. This indicated that the mango ripening method has a greater impact on compounds abundance than the concentration of ethylene used for artificial ripening, but different ethylene concentrations do indeed affect the compound patterns. Among the 69 clustered metabolites, the contents of 22 and 20 metabolites (FC > 2 or FC < 0.5) were higher in the NR group than in AR and CK mangoes, respectively.

### 3.3. Metabolic Pathway Analysis

**Figure 3:**
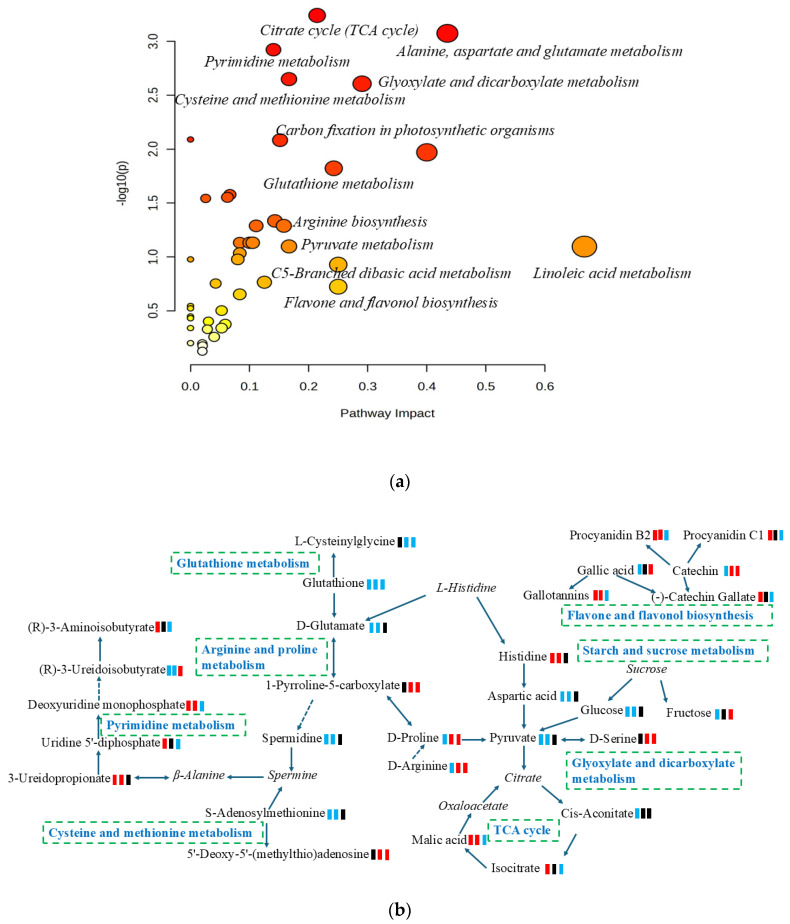
(**a**) Overview of enrichment analysis offers a metabolomics view, which displays matched pathways as circles; (**b**) pathways and relevant metabolites of mango ripened by different methods; green square: down-regulation; red square: up-regulation; black square: no significant change. From left to right, artificially ripened group/naturally ripened group, artificially ripened group/control group, and naturally ripened group/control group. The italics represent metabolites in the pathways but were not detected in this study.

Metabolic pathway analysis is an effective method for examining the direct intrinsic relationships among metabolites and can reconstruct biochemical reaction networks [27]. In this study, a differential metabolite pathway analysis was carried out using metaboanalyst 6.0 software to identify the most relevant metabolic pathways involved in the metabolic responses to mangoes with different ripening methods. As shown in Figure 3a and Table S1, a total of thirty-four pathways were predicted, of which twenty-six pathways had an impact value greater than 0.05. These pathways included citrate cycle (TCA cycle), flavone and flavonol biosynthesis, linoleic acid metabolism, alanine, aspartate and glutamate metabolism, glyoxylate and dicarboxylate metabolism, C5-Branched dibasic acid metabolism, glutathione metabolism, pyruvate metabolism, and starch and sucrose metabolism. Based on the KEGG pathway database, the main pathways of mango metabolism affected by ripening methods are summarized and illustrated in Figure 3b.

### 3.4. Elucidating the Biological Functions of Biomarkers

The maturity of the fruit is a key factor determining its quality [1]. Our research results indicate that different ripening methods lead to changes in the endogenous metabolites of mangoes. Glucose and fructose are important soluble sugars in fruits, and their accumulation contributes to the sweetness of the fruit and enhances its commercial value [10]. The levels of glucose and fructose in the NR group mangoes were significantly higher than that in the AR (9.09 times and 2.33 times, respectively) and CK (1.23 times and 1.69 times, respectively) groups. This is attributed to the fact that sugars, including glucose and fructose, are generated as the end product of photosynthetic activity [28], and the tree-ripened mangoes have the longest exposure to sunlight. Pyruvate can be derived from glucose and is the end product of glycolysis, playing a crucial role in the metabolic connections of carbohydrates, fatty acids, and amino acids [29]. The respiration rate of artificially ripened mangoes is relatively high, accelerating the degradation of pyruvate, which is converted to acetoacetyl-CoA and then further oxidized through the citrate cycle, promoting the production of malic acid and isocitrate. Organic acids are important components of mango flavor. Malic acid and isocitrate are the main organic acids in mangoes. Usually, the malic acid and isocitrate content in post-harvest mangoes significantly decrease along with climacteric changes during the ripening process [30]. Malic acid and isocitrate contents in AR group were 6.08 and 32.98 times higher than those in NR group, respectively, indicating that artificially ripened mangoes may taste slightly more acidic compared to naturally ripened ones.

Increasing evidence has suggested that the accumulation of abscisic acid (ABA) in climacteric fruits is involved in regulating and influencing the fruit ripening process [31]. Naturally ripened mangoes contain higher levels of ABA compared to artificially ripened mangoes. ABA accumulation promotes an increase in sweetness and a decrease in acidity in mangoes, which primarily occurs at the ripening stage and is strongly correlated with the fruit ripening process [32]. In addition, abscisic acid is involved in the biosynthesis of carotenoid. The carotenoid content significantly increases during the ripening process of mango fruit, which is considered an important indicator of the nutritional composition and color of mangoes [33]. The higher accumulation of carotenoids in naturally ripened mangoes makes their epidermis appear brighter yellow than in artificially ripened mangoes. Amino acids are important primary metabolites that serve as the foundation for various biosynthetic pathways, playing a key role in signal transduction and protein synthesis [34]. The contents of D-glutamate, D-arginine, Homo-L-arginine, D-proline, aspartic acid, and 7,8-Diaminopelargonic acid in artificially ripened mangoes are lower than those in naturally ripened mangoes. They contribute to stress responses, plant growth, and the production of flavor compounds in food. Mangoes are rich in vitamins. Niacinamide (Vitamin B3) was found in higher concentrations in naturally ripened mangoes, whereas α-tocopherol (vitamins E) and thiamin (Vitamin B1) were more abundant in artificially ripened mangoes. Ethylene can induce an increase in the expression of p-hydroxyphenylpyruvate dioxygenase, promoting elevated levels of α-tocopherol in mangoes [35]. Since the synthesis of vitamin B is related to the differentiation state of cells, these vitamins are influenced by several pre- and post-harvest factors, as well as the ripening stage [36].

Polyphenols are naturally occurring bioactive compounds that are widely present in mangoes. Our results found that the contents of mangiferin, arbutin, quercetin, catechin, gallic acid, rutin, and rhamnetin were present in higher amounts in naturally ripened mangoes than in artificially ripened mangoes. These important components were considered to possess significant nutritional value and health benefits [37]. For example, mangiferin exhibits antibacterial, anti-allergic, anti-inflammatory, analgesic, anti-atherosclerotic, and immunomodulatory activities [38]. In contrast, the contents of procyanidin B2, procyanidin C1, (-)-catechin gallate, gallotannins, lancerin, and 3,4-Dihydroxybenzaldehyde are higher in artificially ripened mangoes than in naturally ripened ones. Procyanidins, as common secondary metabolites, are one of the most abundant polyphenols in plants. Procyanidin B2 and procyanidin C1 contents in AR group were 3.48 and 4.27 times higher than those in NR group, respectively. They possess strong antioxidant and free radical scavenging activities [39]. Gallotannins also exhibits significant antioxidant properties, helping to protect mango cells from oxidative damage, thereby maintaining fruit freshness and extending shelf life [37]. However, it is associated with antinutritional effects, as higher levels of gallotannins can lead to increased astringency in the fruit, affecting its taste and reducing consumer purchase intent [40]. In summary, different ripening methods have a significant impact on the metabolic components of mangoes.

## 4. Conclusions

The non-targeted metabolomics approach based on UPLC-QTOF combined with chemometric analysis provides an effective method for analyzing the differences in the metabolic composition profiles of mangoes subjected to different ripening methods. PCA of data obtained in both positive and negative ion modes could separate mangoes with different ripening methods. The results indicated significant differences in the metabolite profiles of mangoes among the artificial ripening, natural maturation, and control groups. An OPLS-DA model was established to select biomarkers, which were then matched with a chemical database. A total of 69 metabolites of interest were structurally identified and annotated. These metabolites, including amino acids, carbohydrates, vitamins, organic acids, polyphenols, and terpenoids, are closely related to the taste and nutritional quality of mangoes. We noted that artificially ripened mangoes were inferior to naturally ripened mangoes in terms of taste (sweetness and acidity) and color; however, the levels of certain vitamins and antioxidants were higher in artificially ripened mangoes. In conclusion, our findings indicate significant differences in taste and nutritional quality between artificially ripened mangoes and naturally ripened mangoes, which influence consumer decision-making. Finally, it is recommended to strictly control the concentration of ethephon during the mango ripening process to ensure optimal quality.

## Figures and Tables

**Table 1:** Fold changes, *p* values, and VIP values of metabolites from mango samples ripened by different methods.

Category	Compound	AR/NR				AR/CK				NR-CK			
		FC	*p* Values	VIP	Up or Down	FC	*p* Values	VIP	Up or Down	FC	*p* Values	VIP	Up or Down
amino acids and derivatives	(R)-3-Amino-4-methylpentanoic acid ^a^	7.62	7.22 × 10^−24^	1.31	↑↑	5.4	8.26 × 10^−19^	1.38	↑↑	0.71	1.22 × 10^−2^	0.83	-
	(R)-3-Aminoisobutyrate ^a^	2.53	8.42 × 10^−7^	1.04	↑↑	1.09	6.87 × 10^−1^	0.11	-	0.43	2.31 × 10^−6^	1.22	↓↓
	(R)-3-Ureidoisobutyrate ^a^	0.3	4.51 × 10^−5^	0.9	↓↓	0.51	2.05 × 10^−3^	0.71	↓	1.69	1.67 × 10^−4^	1.14	↑↑
	1-Aminocyclopropane-1-carboxylate	0.95	6.52 × 10^−1^	0.09	-	1.99	1.27 × 10^−4^	0.9	↑	2.1	5.23 × 10^−5^	1.16	↑↑
	1-Pyrroline-5-carboxylate	1.31	3.71 × 10^−3^	0.71	-	3.01	8.13 × 10^−12^	1.31	↑↑	2.3	1.57 × 10^−1^	0.53	↑↑
	2-Aminobenzoic acid ^a^	3.4	2.49 × 10^−15^	1.27	↑↑	2.25	4.21 × 10^−11^	1.27	↑↑	0.66	3.95 × 10^−3^	0.91	↓
	3-Ureidopropionate	4	3.02 × 10^−4^	0.86	↑↑	4.39	1.16 × 10^−4^	1.01	↑↑	1.1	3.56 × 10^−1^	0.41	-
	7,8-Diaminopelargonic acid ^a^	0.43	2.64 × 10^−6^	0.99	↓↓	1.58	1.20 × 10^−2^	0.72	↑	3.67	2.34 × 10^−9^	1.4	↑↑
	Aspartic acid	0.27	3.02 × 10^−8^	1.1	↓↓	0.32	4.27 × 10^−7^	1.1	↓↓	1.21	8.22 × 10^−2^	0.61	-
	D-Arginine ^a^	0.39	2.60 × 10^−4^	0.84	↓↓	1.64	5.62 × 10^−1^	0.2	↑	4.24	6.20 × 10^−5^	1.16	↑↑
	D-Glutamate ^a^	0.41	4.61 × 10^−8^	1.1	↓↓	0.6	4.60 × 10^−5^	1	↓	1.44	2.76 × 10^−5^	1.2	-
	D-Lysine ^a^	3.35	1.24 × 10^−11^	1.21	↑↑	4.86	1.29 × 10^−14^	1.3	↑↑	1.45	1.51 × 10^−5^	1.17	-
	D-Proline	0.71	1.73 × 10^−3^	0.76	-	2.9	4.42 × 10^−9^	1.2	↑↑	4.12	2.76 × 10^−9^	1.38	↑↑
	D-Serine	1.48	3.26 × 10^−2^	0.51	-	2.97	1.60 × 10^−7^	1.13	↑↑	2.01	2.89 × 10^−5^	1.22	↑↑
	Histidine	2.65	1.11 × 10^−12^	1.23	↑↑	3.27	2.69 × 10^−16^	1.37	↑↑	1.24	1.05 × 10^−1^	0.61	-
	Homo-L-arginine	0.82	9.22 × 10^−2^	0.43	-	1.88	6.84 × 10^−8^	1.18	↑	2.3	1.84 × 10^−6^	1.31	↑↑
	L-Isoleucine	1.9	3.46 × 10^−3^	0.68	↑	3.97	1.27 × 10^−7^	1.13	↑↑	2.08	4.34 × 10^−4^	1.09	↑↑
	S-Adenosylmethionine	0.5	3.55 × 10^−6^	1.08	↓↓	0.54	3.26 × 10^−5^	0.95	↓	1.05	5.57 × 10^−1^	0.17	-
carbohydrates and derivatives	Fructose	0.43	2.23 × 10^−5^	1.03	↓↓	0.73	2.53 × 10^−2^	0.53	-	1.69	3.86 × 10^−7^	1.34	↑
	Glucose	0.11	7.27 × 10^−10^	1.17	↓↓	0.13	1.90 × 10^−11^	1.3	↓↓	1.23	7.05 × 10^−1^	0.06	-
lipid compounds	14,15-Dehydrocrepenynic acid	0.4	1.55 × 10^−7^	1.1	↓↓	0.5	4.88 × 10^−1^	0.15	↓↓	1.25	8.74 × 10^−2^	0.64	-
	linoleic acid	3.34	6.01 × 10^−7^	1.07	↑↑	2.3	4.47 × 10^−6^	1.03	↑↑	0.69	6.66 × 10^−2^	0.58	-
	Monopalmitolein (9c)	0.27	3.01 × 10^−7^	1.08	↓↓	0.35	5.99 × 10^−9^	1.25	↓↓	1.29	5.18 × 10^−1^	0.13	-
	Phytosphingosine	0.79	3.87 × 10^−2^	0.48	-	0.5	2.05 × 10^−7^	1.09	↓↓	0.65	3.08 × 10^−5^	1.2	↓
	Sphinganine	0.5	6.83 × 10^−5^	0.88	↓↓	0.5	3.60 × 10^−7^	1.09	↓↓	0.93	5.20 × 10^−1^	0.22	-
nucleosides	5’-Deoxy-5’-(methylthio)adenosine ^a^	0.8	4.97 × 10^−2^	0.47	-	1.66	3.53 × 10^−4^	0.92	↑	2.07	8.03 × 10^−7^	1.3	↑↑
	Deoxyuridine monophosphate	3.8	4.26 × 10^−14^	1.26	↑↑	1.8	8.13 × 10^−8^	1.19	↑	0.47	4.43 × 10^−5^	1.16	↓↓
	Uridine 5-diphosphate	2.98	1.71 × 10^−8^	1.13	↑↑	1.2	4.92 × 10^−3^	0.71	-	0.4	1.09 × 10^−4^	1.15	↓↓
organic acids	2,5-Furandicarboxylic acid	0.38	1.17 × 10^−4^	1.03	↓↓	0.5	2.55 × 10^−3^	0.86	↓↓	1.36	1.91 × 10^−3^	1.21	-
	2-Carboxy-2,5-dihydro-5-oxofuran-2-acetate	4.33	4.82 × 10^−15^	1.26	↑↑	2.91	3.13 × 10^−9^	1.21	↑↑	0.67	7.35 × 10^−2^	0.6	-
	3-Coumaric acid	21.5	8.31 × 10^−17^	1.28	↑↑	14.22	2.63 × 10^−15^	1.34	↑↑	0.66	3.47 × 10^−5^	1.17	↓
	Benzoic acid ^a^	1.07	1.97 × 10^−1^	0.37	-	4.67	7.73 × 10^−23^	1.38	↑↑	4.37	3.53 × 10^−12^	1.39	↑↑
	Cis-Aconitate	0.5	3.73 × 10^−4^	0.83	↓↓	0.84	1.34 × 10^−1^	0.39	-	1.4	1.42 × 10^−3^	1	-
	Isocitrate	32.98	1.77 × 10^−12^	1.23	↑↑	0.95	4.88 × 10^−1^	0.25	-	0.03	4.62 × 10^−12^	1.41	↓↓
	Malic acid	6.08	6.18 × 10^−7^	1.05	↑↑	1.6	1.00 × 10^−2^	0.72	↑	0.26	6.65 × 10^−4^	1.03	↓↓
	Pyruvate	0.5	2.08 × 10^−3^	1.06	↓↓	0.69	7.80 × 10^−3^	0.7	-	1.29	3.53 × 10^−1^	0.31	-
	Quinic acid	27.34	1.24 × 10^−16^	1.28	↑↑	14.23	1.47 × 10^−14^	1.34	↑↑	0.52	4.54 × 10^−7^	1.28	↓
others	(1R,6S)-1,6-Dihydroxycyclohexa-2,4-diene-1-carboxylate	3.86	5.17 × 10^−13^	1.24	↑↑	2.8	2.99 × 10^−10^	1.26	↑↑	0.73	8.18 × 10^−2^	0.53	-
	5-Dehydro-4-deoxy-D-glucarate	0.5	1.97 × 10^−4^	0.86	↓↓	0.8	7.91 × 10^−2^	0.46	-	1.4	1.12 × 10^−3^	1.02	-
	Abscisic acid	0.5	4.24 × 10^−5^	0.91	↓↓	0.44	1.16 × 10^−7^	1.16	↓↓	0.81	7.75 × 10^−2^	0.57	-
	Isopentenyladenine-9-N-glucoside	22.32	5.61 × 10^−17^	1.29	↑↑	30.21	5.00 × 10^−19^	1.39	↑↑	1.35	1.73 × 10^−1^	0.51	-
peptides	Glutathione	0.11	1.79 × 10^−1^	0.34	↓↓	0.02	3.74 × 10^−14^	1.34	↓↓	0.19	3.69 × 10^−4^	1.08	↓↓
	L-Cysteinylglycine	0.85	1.45 × 10^−1^	0.38	-	0.12	1.69 × 10^−7^	1.18	↓↓	0.15	1.69 × 10^−4^	1.11	↓↓
phospholipids	Glycerophosphocholine	0.62	5.26 × 10^−4^	0.87	↓	0.5	7.64 × 10^−9^	1.26	↓↓	0.93	3.48 × 10^−1^	0.34	-
	LPC(1-acyl 16:1)	0.16	1.14 × 10^−12^	1.25	↓↓	0.59	1.49 × 10^−6^	1.12	↓	3.65	2.18 × 10^−7^	1.28	↑↑
	LPC(1-acyl 18:1)	0.3	2.03 × 10^−10^	1.21	↓↓	0.71	1.86 × 10^−7^	1.15	-	2.4	4.32 × 10^−5^	1.15	↑↑
	LPC(1-acyl 18:3)	0.69	1.70 × 10^−2^	0.63	-	0.83	2.12 × 10^−2^	1.08	-	1.2	4.27 × 10^−1^	0.29	-
polyphenols	(-)-Catechin Gallate	3.92	3.80 × 10^−10^	1.17	↑↑	1.35	3.27 × 10^−2^	0.53	-	0.34	2.05 × 10^−7^	1.29	↓↓
	3,4-Dihydroxybenzaldehyde	3.4	1.35 × 10^−9^	1.16	↑↑	1.95	4.43 × 10^−5^	1	↑	0.57	9.33 × 10^−2^	0.5	↓
	3,4’-Isopropylidenediphenol	0.21	8.57 × 10^−16^	1.27	↓↓	0.16	4.11 × 10^−19^	1.37	↓↓	0.76	9.07 × 10^−4^	1.02	-
	Arbutin	0.49	7.38 × 10^−5^	1.03	↓↓	0.84	4.34 × 10^−1^	0.25	-	1.53	2.70 × 10^−2^	0.78	↑
	Calycosin-7-o-glucoside	1.48	5.90 × 10^−3^	0.66	-	3.08	3.43 × 10^−6^	1.07	↑↑	2.08	4.73 × 10^−1^	0.36	↑↑
	Catechin	0.3	4.89 × 10^−6^	1.39	↓↓	2.14	2.71 × 10^−3^	0.77	↑↑	7.1	2.63 × 10^−8^	1.34	↑↑
	Gallic acid	0.41	7.49 × 10^−2^	0.42	↓↓	1.17	6.14 × 10^−1^	0.09	-	2.85	9.38 × 10^−2^	1.08	↑↑
	Gallotannins	24.27	6.45 × 10^−17^	1.28	↑↑	14.35	2.84 × 10^−15^	1.34	↑↑	0.59	5.25 × 10^−5^	1.14	↓
	Lancerin	4.17	2.13 × 10^−7^	1.1	↑↑	1.35	8.50 × 10^−2^	0.55	-	0.32	7.78 × 10^−4^	0.99	↓↓
	Mangiferin	0.37	7.00 × 10^−5^	1.01	↓↓	0.66	6.20 × 10^−3^	0.76	↓	1.79	3.04 × 10^−2^	0.74	↑
	Procyanidin B2	3.48	1.36 × 10^−7^	1.08	↑↑	1.57	5.34 × 10^−3^	0.69	↑	0.45	5.70 × 10^−4^	1.01	↓↓
	Procyanidin c1	4.27	4.55 × 10^−8^	1.11	↑↑	1.03	8.75 × 10^−1^	0.07	-	0.24	9.86 × 10^−6^	1.14	↓↓
	Quercetin	0.44	1.82 × 10^−5^	0.94	↓↓	0.42	6.40 × 10^−6^	1.02	↓↓	0.95	5.33 × 10^−1^	0.27	-
	Rhamnetin	0.39	3.86 × 10^−8^	1.09	↓↓	2.61	3.77 × 10^−7^	1.13	↑↑	6.61	3.68 × 10^−11^	1.43	↑↑
	Rutin	0.4	5.11 × 10^−4^	0.8	↓↓	1.12	9.90 × 10^−2^	0.51	-	2.81	8.06 × 10^−3^	1.1	↑↑
	Tricin 5-O-hexoside	1.45	8.48 × 10^−3^	0.64	-	3.01	8.29 × 10^−6^	1.05	↑↑	2.08	4.73 × 10^−1^	0.36	↑↑
terpenoids	Carotenoid	0.27	7.19 × 10^−11^	1.18	↓↓	0.6	1.27 × 10^−8^	1.2	↓	2.22	1.00 × 10^−4^	1.08	↑↑
	Pogostone	1.92	1.55 × 10^−7^	1.08	↑	0.87	1.18 × 10^−1^	0.46	-	0.45	4.44 × 10^−7^	1.29	↓↓
	(-)-Caryophyllene oxide	3.44	6.02 × 10^−13^	1.24	↑↑	2.07	7.18 × 10^−9^	1.21	↑↑	0.6	3.75 × 10^−4^	1.03	↓
vitamins with cofactors	alpha-Tocopherol	7.43	5.29 × 10^−9^	1.15	↑↑	2.25	6.22 × 10^−3^	0.7	↑↑	0.3	9.03 × 10^−6^	1.23	↓↓
	Niacinamide ^a^	0.39	1.66 × 10^−5^	0.93	↓↓	2.55	5.76 × 10^−4^	0.9	↑↑	6.57	8.77 × 10^−12^	1.38	↑↑
	Thiamin	11.16	1.22 × 10^−9^	1.16	↑↑	2.8	1.80 × 10^−3^	0.78	↑↑	0.25	6.13 × 10^−6^	1.26	↓↓

FC fold changes; ↑ up regulated; ↑↑ up regulated with fold change over 2; ↓ down regulated, ↓↓ down regulated with fold change over 2. AR artificially ripened group; NR naturally ripened group; CK control group. ^a^ identified using MS/MS high-resolution library.

## Data Availability

The original contributions presented in the study are included in the article/Supplementary Material, further inquiries can be directed to the corresponding authors.

## Supplementary Materials

The following supporting information can be downloaded at: https://www.mdpi.com/article/10.3390/foods13223548/s1, Figure S1: (a) OPLS-DA score plot of artificially ripened group and control group; (b) OPLS-DA model of artificially ripened group and control group (one predictive and one orthogonal components); (c) OPLS-DA score plot of naturally ripened group and control group; (d) OPLS-DA model of naturally ripened group and control group (one predictive and one orthogonal components). The colored ellipses represent 95% confidence regions for each group.; Figure S2: Permutation tests plot of OPLS-DA model for mango ripened by different methods ((a), artificially ripened group and naturally ripened group; (b), artificially ripened group and control group; (c), naturally ripened group and control group).; Figure S3: Extracted ion chromatogram, isotope pattern and MS/MS spectrum against library of the identified metabolites.; Table S1: List of metabolic pathway by enrichment analysis for the metabolites.

## References

[1] Sivakumar D., Jiang Y., Yahia E.M. (2011). Maintaining mango (*Mangifera indica* L.) fruit quality during the export chain. Food Res. Int..

[2] Zhang Z., Zhu Q., Hu M., Gao Z., An F., Li M., Jiang Y. (2017). Low-temperature conditioning induces chilling tolerance in stored mango fruit. Food Chem..

[3] Food and Agriculture Organization of the United Nations Data Collection. https://www.fao.org/faostat/en/#data/QCL.

[4] Joas J., Vulcain E., Desvignes C., Morales E., Léchaudel M. (2011). Physiological age at harvest regulates the variability in postharvest ripening, sensory and nutritional characteristics of mango (*Mangifera indica* L.) cv. Coghshall due to growing conditions. J. Sci. Food Agric..

[5] Cortés V., Ortiz C., Aleixos N., Blasco J., Cubero S., Talens P. (2016). A new internal quality index for mango and its prediction by external visible and near-infrared reflection spectroscopy. Postharvest Biol. Technol..

[6] Ramayya N., Niranjan K., Duncan E. (2012). Effects of modified atmosphere packaging on quality of ‘Alphonso’ Mangoes. J. Food Sci. Technol..

[7] Ullah H., Ahmad S., Thompson A.K., Anwar R., Nafees M. (2010). Effect of ‘oxygen and carbon-dioxide’ on the post-harvest management in tree-ripe mango storage. J. Chem. Soc. Pakistan..

[8] Singh Z., Singh R.K., Sane V.A., Nath P. (2013). Mango-Postharvest Biology and Biotechnology. Crit. Rev. Plant Sci..

[9] Paul V., Pandey R., Srivastava G.C. (2011). The fading distinctions between classical patterns of ripening in climacteric and non-climacteric fruit and the ubiquity of ethylene—An overview. J. Food Sci. Technol..

[10] Liu B., Xin Q., Zhang M., Chen J., Lu Q., Zhou X., Li X., Zhang W., Feng W., Pei H. (2022). Research progress on mango post-harvest ripening physiology and the regulatory technologies. Foods.

[11] Ho B.T., Hofman P.J., Joyce D.C., Bhandari B.R. (2016). Uses of an innovative ethylene-α-cyclodextrin inclusion complex powder for ripening of mango fruit. Postharvest Biol. Technol..

[12] Razzaq K., Singh Z., Khan A.S., Khan S.A.K.U., Ullah S. (2016). Role of 1-MCP in regulating ‘Kensington Pride’ mango fruit softening and ripening. Plant Growth Regul..

[13] Pino J.A., Mesa J. (2006). Contribution of volatile compounds to mango (*Mangifera indica* L.) aroma. Flavour Fragr. J..

[14] Zaharah S.S., Singh Z., Symons G.M., Reid J.B. (2013). Mode of action of abscisic acid in triggering ethylene biosynthesis and softening during ripening in mango fruit. Postharvest Biol. Technol..

[15] Scalbert A., Brennan L., Fiehn O., Hankemeier T., Kristal B.S., van Ommen B., Pujos-Guillot E., Verheij E., Wishart D., Wopereis S. (2009). Mass-spectrometry-based metabolomics: Limitations and recommendations for future progress with particular focus on nutrition research. Metabolomics.

[16] Shen B., Yi X., Sun Y., Bi X., Du J., Zhang C., Quan S., Zhang F., Sun R., Qian L. (2020). Proteomic and Metabolomic Characterization of COVID-19 Patient Sera. Cell.

[17] He Z., Wang Y., Zhang Y., Cheng H., Liu X. (2018). Stereoselective bioaccumulation of chiral PCB 91 in earthworm and its metabolomic and lipidomic responses. Environ Pollut..

[18] Dudka I., Kossowska B., Senhadri H., Latajka R., Hajek J., Andrzejak R., Antonowicz-Juchniewicz J., Gancarz R. (2014). Metabonomic analysis of serum of workers occupationally exposed to arsenic, cadmium and lead for biomarker research: A preliminary study. Environ Int..

[19] Wang J., Zhao L., Liu X., He Z. (2022). Stereoselective effects of chiral epoxiconazole on the metabolomic and lipidomic profiling of leek. Food Chem..

[20] Broeckling C.D., Hoyes E., Richardson K., Brown J.M., Prenni J.E. (2018). Comprehensive Tandem-Mass-Spectrometry Coverage of Complex Samples Enabled by Data-Set-Dependent Acquisition. Anal. Chem..

[21] He G., Chen X., Hou X., Yu X., Han M., Qiu S., Li Y., Qin S., Wang F. (2023). UPLC-Q-TOF/MS-based metabolomic analysis reveals the effects of asomate on the citrus fruit. Curr. Res. Food Sci..

[22] Li M., Li L., Kong Z., Gregoire N., Quan R., Luo Z., Lin X., Simal-Gandara J., Fan B., Wang F. (2023). Integrative analysis of metabolome and genome-wide transcriptome reveal the flavor changes in apple (*Malus pumila* Mill) after the novel acaricide cyflumetofen application. LWT-Food Sci. Technol..

[23] Zhang X., Andrew P.B., Mishchuk D.O., Fake C.E., O’Mahony M.A., Slupsky C.M. (2012). Fertilisation and pesticides affect mandarin orange nutrient composition. Food Chem..

[24] Liu Y., Liu R., Yu S., Diao J., Deng Y., Zheng M., Nie Y., Li J.Q., Pan C., Zhou Z. (2023). Insights into the Mechanism of Flavor Loss in Strawberries Induced by Two Fungicides Integrating Transcriptome and Metabolome Analysis. J. Agric. Food Chem..

[25] Gika H.G., Theodoridis G.A., Wingate J.E., Wilson I.D. (2007). Within-Day Reproducibility of an HPLC?MS-Based Method for Metabonomic Analysis:? Application to Human Urine. J. Proteome Res..

[26] Franceschi P., Masuero D., Vrhovsek U., Mattivi F., Wehrens R. (2012). A benchmark spike-in data set for biomarker identification in metabolomics. J Chemometr..

[27] Klamt S., Stelling J. (2003). Two approaches for metabolic pathway analysis?. Trends Biotechnol..

[28] Stein O., Granot D. (2019). An overview of sucrose synthases in plants. Front. Plant Sci..

[29] Suh J.H., Madden R.T., Sung J., Chambers A.H., Crane J., Wang Y. (2022). Pathway-Based Metabolomics Analysis Reveals Biosynthesis of Key Flavor Compounds in Mango. J. Agric. Food Chem..

[30] Nordey T., Léchaudel M., Génard M., Joas J. (2014). Spatial and temporal variations in mango colour, acidity, and sweetness in relation to temperature and ethylene gradients within the fruit. J. Plant Physiol..

[31] Qiao H., Zhang H., Wang Z., Shen Y. (2021). Fig fruit ripening is regulated by the interaction between ethylene and abscisic acid. J. Integr. Plant Biol..

[32] Wu S., Wu D., Song J., Zhang Y., Tan Q., Yang T., Yang J., Wang S., Xu J., Xu W. (2022). Metabolomic and transcriptomic analyses reveal new insights into the role of abscisic acid in modulating mango fruit ripening. Hortic. Res.-England.

[33] Dellapenna D., Pogson B.J. (2006). Vitamin synthesis in plants: Tocopherols and carotenoids. Annu. Rev. Plant Biol..

[34] Hildebrandt T.M., Nesi A.N., Araújo W.L., Braun H.P. (2015). Amino acid catabolism in plants. Mol. Plant.

[35] Singh R.K., Ali S.A., Nath P., Sane V.A. (2011). Activation of ethylene-responsive p-hydroxyphenylpyruvate dioxygenase leads to increased tocopherol levels during ripening in mango. J. Exp. Bot..

[36] Aslam J., Mujib A., Fatima Z., Sharma M.P. (2010). Variations in vinblastine production at different stages of somatic embryogenesis, embryo, and field-grown plantlets of *Catharanthus roseus* L. (G) Don, as revealed by HPLC. Vitr. Cell. Dev. Biol. Plant.

[37] Maldonado-Celis M.E., Yahia E.M., Bedoya R., Patricia L., Loango N., Johanny A., Restrepo B., Ospina J.C.G. (2019). Chemical composition of mango (*Mangifera indica* L.) fruit: Nutritional and phytochemical compounds. Front. Plant Sci..

[38] Imran M., Arshad M.S., Butt M.S., Kwon J.-H., Arshad M.U., Sultan M.T. (2017). Mangiferin: A natural miracle bioactive compound against lifestyle related disorders. Lipids Health Dis..

[39] Yang H., Tuo X., Wang L., Tundis R., Portillo M.P., Gandara J.S., Yu Y., Zou L., Xiao J., Deng J. (2021). Bioactive procyanidins from dietary sources: The relationship between bioactivity and polymerization degree. Trends Food Sci Technol..

[40] Engels C., Gänzle M.G., Schieber A. (2012). Fast LC–MS analysis of gallotannins from mango (*Mangifera indica* L.) kernels and effects of methanolysis on their antibacterial activity and iron binding capacity. Food Res. Int..

