# Supplementary material for: Liquid Chromatography Quadrupole Time-of-Flight Mass Spectrometry-Based Metabolic Characterization of Mango Ripened by Different Methods
Source: Foods. 2024 Nov 7;13(22):3548. doi: 10.3390/foods13223548 (PMC11593038; doi:10.3390/foods13223548)
Supplement: Supplementary file 1 [file foods-13-03548-s001.zip › foods-3278538-supplementary.pdf]

Liquid chromatography quadrupole time-of-flight mass spectrometry-based metabolic characterization of mango ripened by different methods

Jishi Wang[1], Chaoqi Ren[2], Jiafu Wang[1], Jiqiang Fu[1], Qingchun Yin[3] Yongping Huang[3*] Zeying He[1*]

1 Key Laboratory for Environmental Factors Control of Agro-product Quality Safety, Ministry of Agriculture, Agro-Environmental Protection Institute, Ministry of Agriculture and Rural Affairs, Tianjin 300191, China; wangjishi@caas.cn (J.W.); wjf199809@163.com (J.W.); 82101225353@caas.cn (J.F.)

2 College of Animal Science and Veterinary Medicine, Tianjin Agricultural University, Tianjin 300384, China; renchaoqi92@163.com

3 Key Laboratory of Tropical Fruits and Vegetables Quality and Safety, Institute of Food Testing, Hainan Acad-emy of Inspection and Testing, State Administration for Market Regulation, Haikou 570311, China; yin-qingchun@163.com

* Correspondence: hyp16210@163.com (Y.H.); hezeying@caas.cn (Z.H.); Tel./Fax: +86-0898-68683062 (Y.H.);

+86-022-23611009 (Z.H.); Fax: +86-022-23611160 (Z.H.)

*Corresponding author:

Yongping Huang, Key Laboratory of Tropical Fruits and Vegetables Quality and Safety, Institute of Food Testing, Hainan Acad-emy of Inspection and Testing, State Administration for Market Regulation, Haikou 570311, China;

Tel.: +86 0898-68683062; Fax: +86 0898-68683062

E-mail: hyp16210@163.com

Zeying He, Key Laboratory for Environmental Factors Control of Agro-product Quality Safety, Ministry of Agriculture, Agro-Environmental Protection Institute, Ministry of Agriculture and Rural Affairs, Tianjin 300191, P.R. China;

Tel.: +86 022-23611009; Fax: +86 022-23611160

E-mail: hezeying@cass.cn

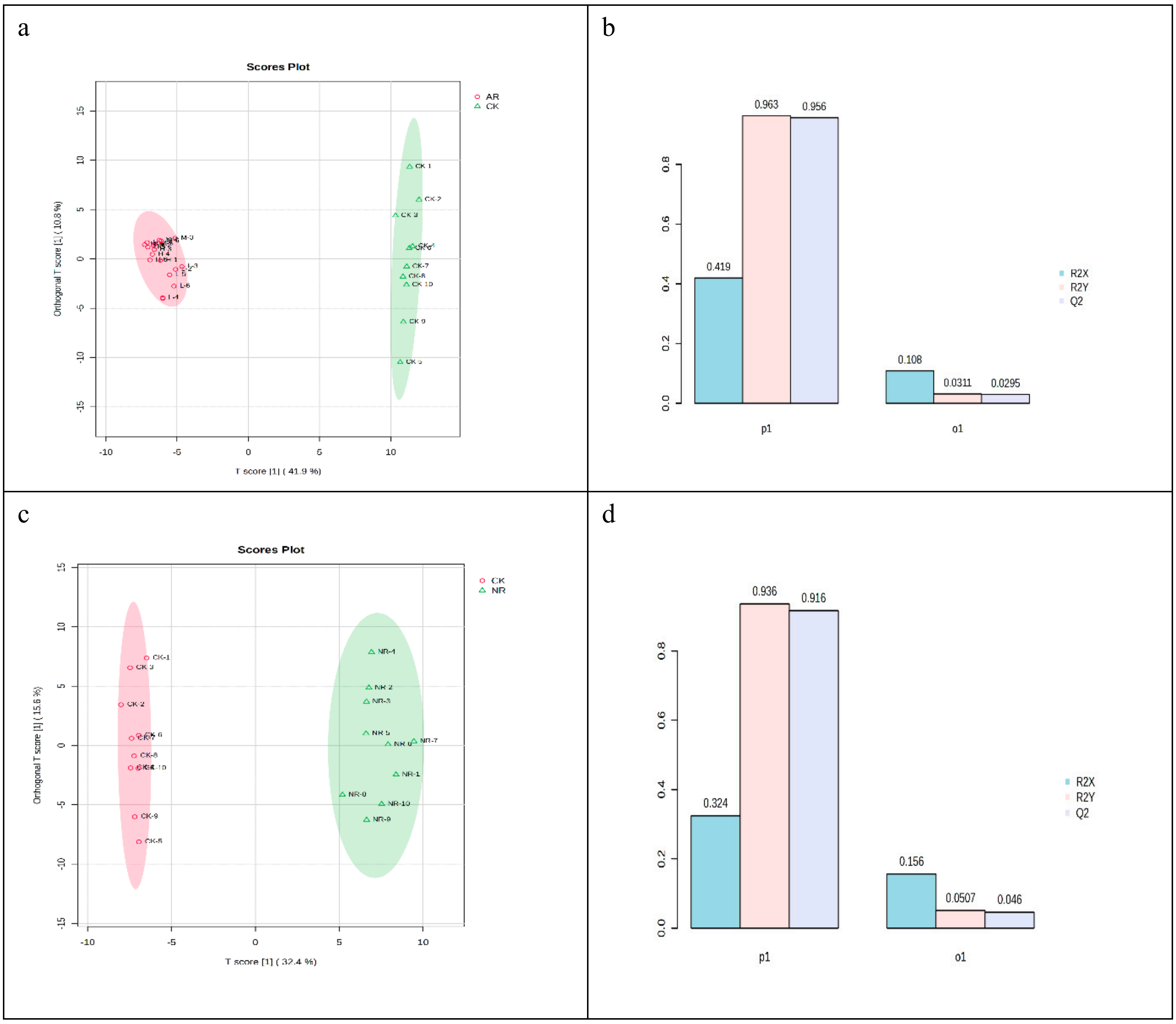

Figure S1 (a) OPLS-DA score plot of artificially ripened group and control group; (b) OPLS-DA model of artificially ripened group and control group (one predictive and one orthogonal components); (c) OPLS-DA score plot of naturally ripened group and control group; (d) OPLS-DA model of naturally ripened group and control group (one predictive and one orthogonal components). The colored ellipses represent 95% confidence regions for each group.

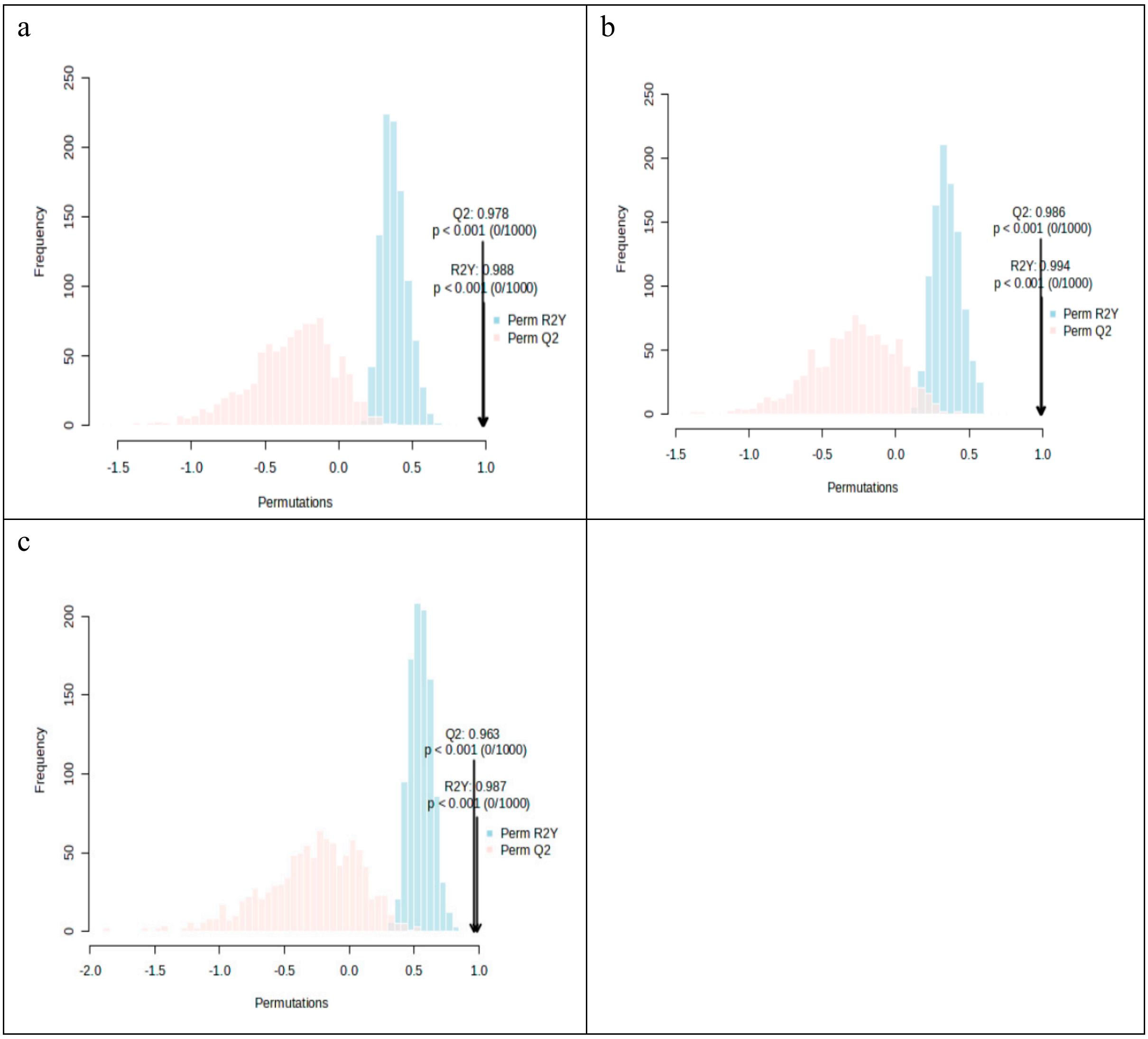

Figure S2 Permutation tests plot of OPLS-DA model for mango ripened by different methods (a, artificially ripened group and naturally ripened group; b, artificially ripened group and control group; c, naturally ripened group and control group)

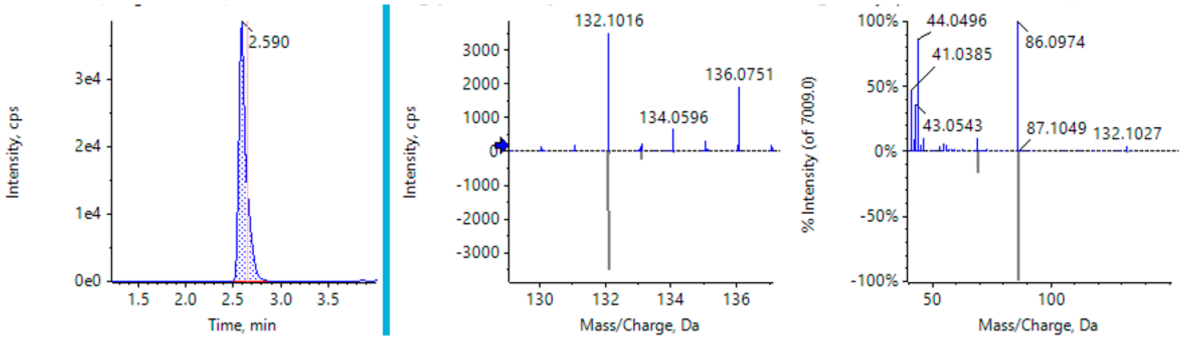

(R)-3-Amino-4-methylpentanoic acid

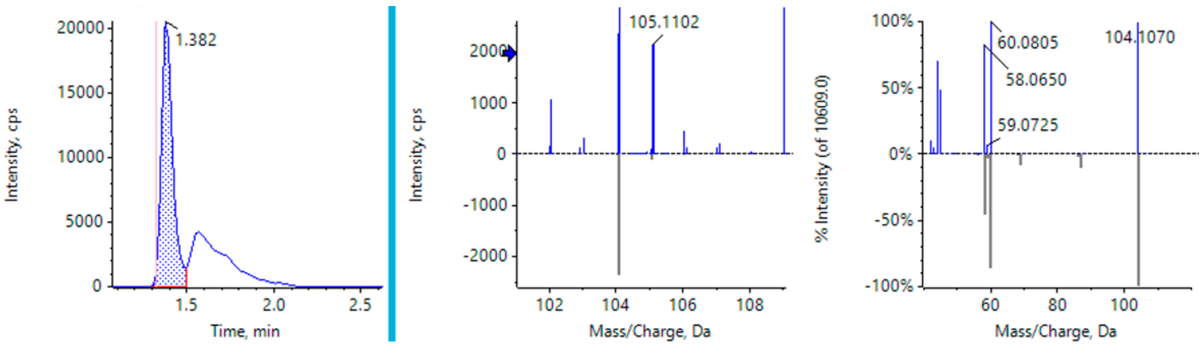

(R)-3-Aminoisobutyrate

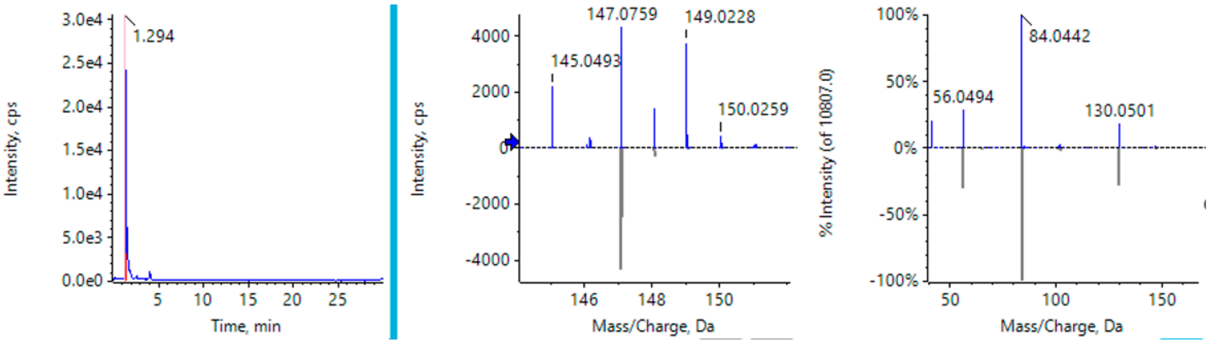

(R)-3-Ureidoisobutyrate

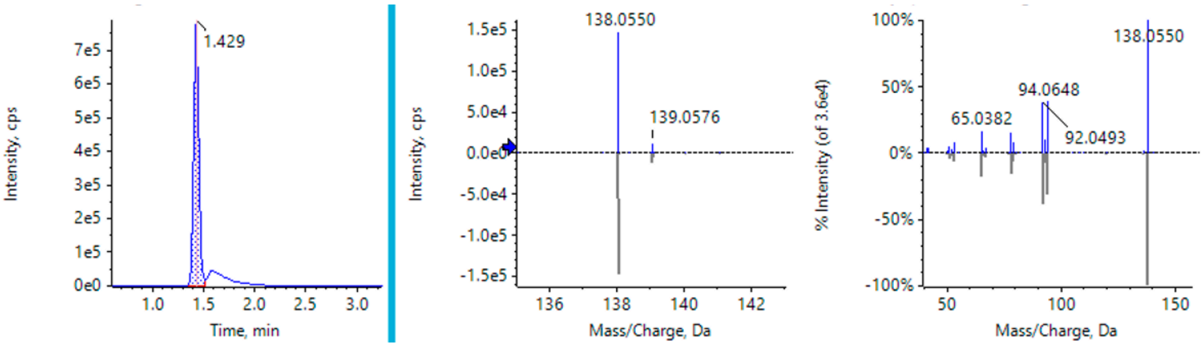

2-Aminobenzoic acid

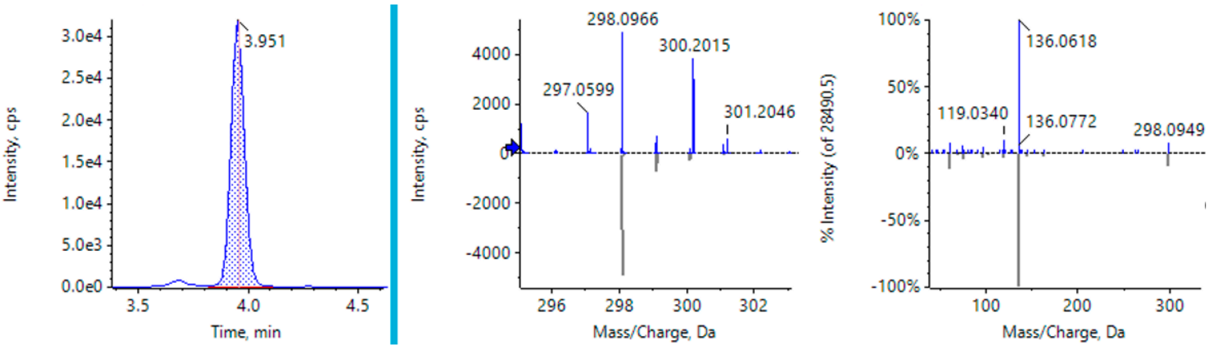

5'-Deoxy-5'-(methylthio)adenosine

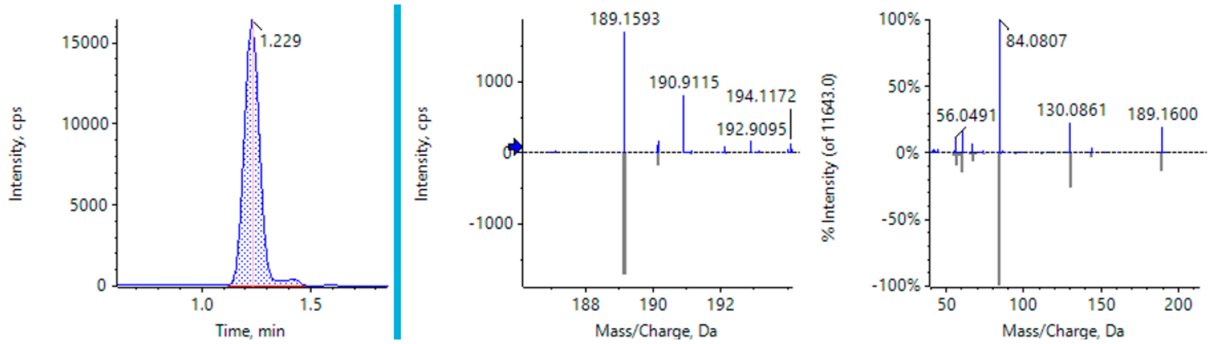

7,8-Diaminopelargonic acid(DAPA)

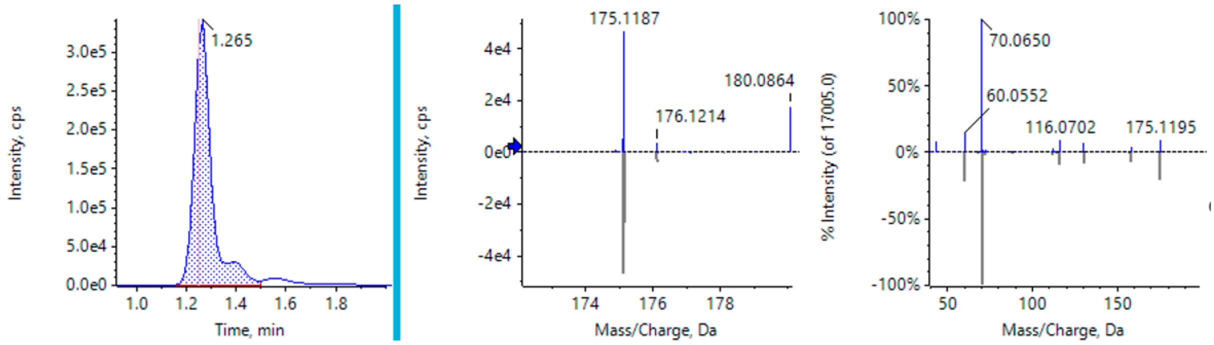

D-Arginine

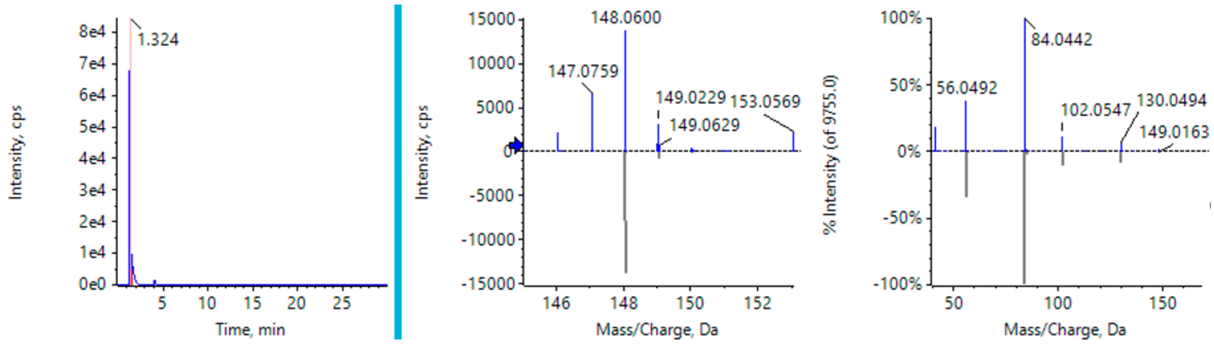

D-Glutamate

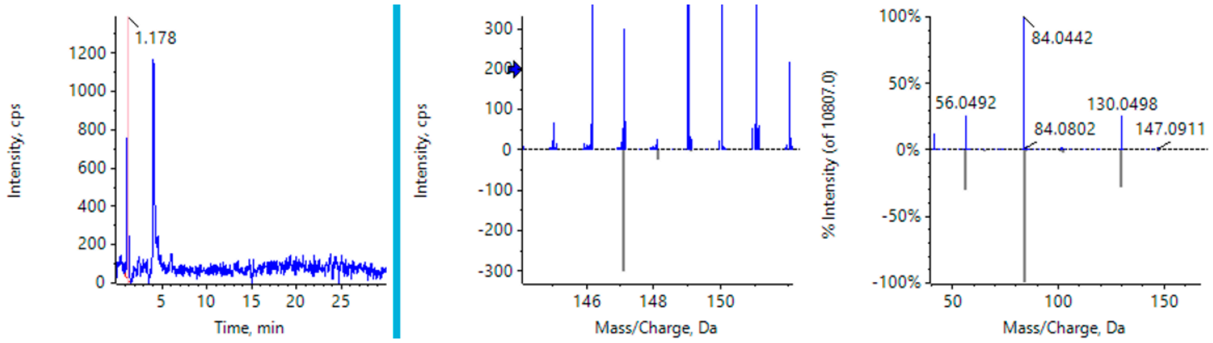

D-Lysine

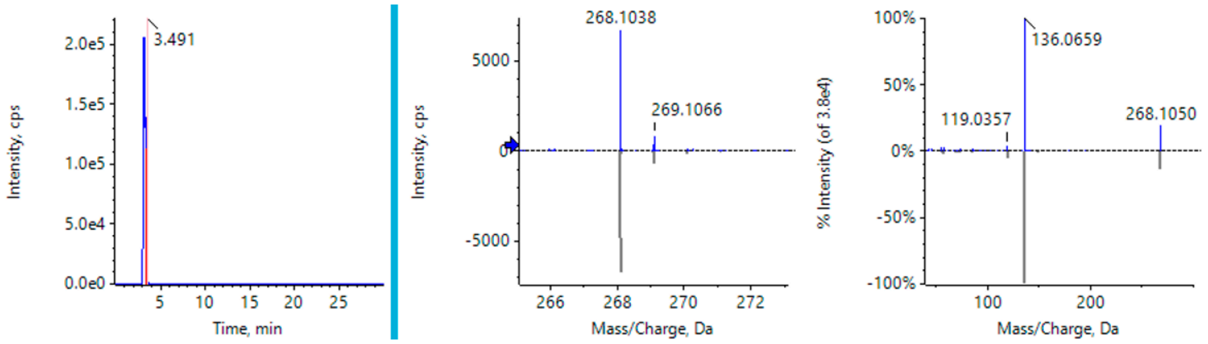

Niacinamide

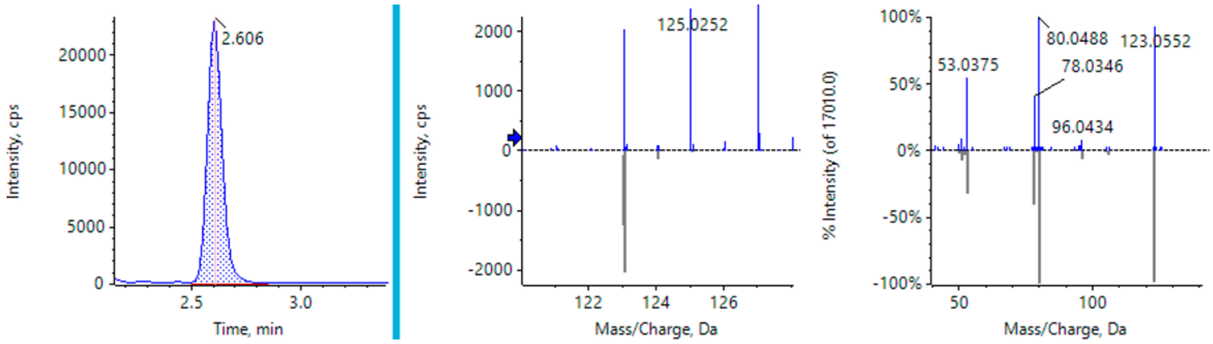

Benzoic acid

Figure S3 Extracted ion chromatogram, isotope pattern and MS/MS spectrum against library of the identified metabolites

Table S1 List of metabolic pathway by enrichment analysis for the metabolites.

Pathway name	Total	hits	Raw P	-Log(P)	FDR	Impact
Linoleic acid metabolism	4	1	0.0802	1.0957	0.365	0.667
Alanine, aspartate and glutamate metabolism	22	4	0.0008	3.0740	0.036	0.435
Glyoxylate and dicarboxylate metabolism	29	4	0.0025	2.6067	0.045	0.290
C5-Branched dibasic acid metabolism	6	1	0.1180	0.9283	0.447	0.250
Flavone and flavonol biosynthesis	10	1	0.1890	0.7236	0.637	0.250
Glutathione metabolism	26	3	0.0151	1.8220	0.152	0.242
Citrate cycle (TCA cycle)	20	4	0.0006	3.2403	0.036	0.214
Pyruvate metabolism	23	2	0.0799	1.0973	0.365	0.167
Cysteine and methionine metabolism	47	5	0.0022	2.6493	0.045	0.167
Arginine biosynthesis	18	2	0.0515	1.2882	0.312	0.158
Carbon fixation in photosynthetic organisms	21	3	0.0083	2.0827	0.107	0.152
Butanoate metabolism	17	2	0.0464	1.3338	0.312	0.143
Pyrimidine metabolism	41	5	0.0012	2.9222	0.036	0.140
Lysine biosynthesis	9	1	0.1718	0.7651	0.618	0.125
beta-Alanine metabolism	18	2	0.0515	1.2882	0.312	0.111
Thiamine metabolism	22	2	0.0739	1.1314	0.365	0.105
Valine, leucine and isoleucine biosynthesis	22	2	0.0739	1.1314	0.365	0.100
Starch and sucrose metabolism	22	2	0.0739	1.1314	0.365	0.083

Pantothenate and CoA biosynthesis	25	2	0.0924	1.0343	0.400	0.083
Nitrogen metabolism	12	1	0.2224	0.6529	0.723	0.083
Sphingolipid metabolism	27	2	0.1054	0.9771	0.417	0.080
Arginine and proline metabolism	32	3	0.0264	1.5778	0.217	0.067
Nicotinate and nicotinamide metabolism	13	2	0.0280	1.5530	0.217	0.063
Glycolysis / Gluconeogenesis	26	1	0.4217	0.3750	1.000	0.059
Fructose and mannose metabolism	18	1	0.3148	0.5019	0.924	0.053
Biosynthesis of various plant secondary metabolites	29	1	0.4574	0.3397	1.000	0.053
Valine, leucine and isoleucine degradation	37	2	0.1765	0.7533	0.618	0.043
Glycerophospholipid metabolism	38	1	0.5523	0.2578	1.000	0.040
Lipoic acid metabolism	24	1	0.3966	0.4017	1.000	0.030
Terpenoid backbone biosynthesis	30	1	0.4688	0.3290	1.000	0.029
Glycine, serine and threonine metabolism	33	3	0.0287	1.5425	0.217	0.026
Amino sugar and nucleotide sugar metabolism	52	1	0.6688	0.1747	1.000	0.020
Porphyrin metabolism	48	1	0.6389	0.1946	1.000	0.020
Glucosinolate biosynthesis	65	1	0.7503	0.1248	1.000	0.020
